# How to Extinguish Fire

**Published:** 1891-09

**Authors:** 


### How to Extinguish Fire.

Take twenty pounds of common salt and ten pounds of sal ammoniac (muriate of ammonia, to be had of any druggist), and dissolve in seven gallons of water. When dissolved it can be bottled, and kept in each room in the house to be used in an emergency. In case of a fire occuring one or two bottles should be immediately thrown with force into the burning place so as to break them and the fire will certainly be extinguished. This is an exceedingly simple process and certainly worth a trial—[*Annals of Hygiene.*

